# N-Terminal Pfs230 Domain Produced in Baculovirus as a Biological Active Transmission-Blocking Vaccine Candidate

**DOI:** 10.1128/CVI.00140-17

**Published:** 2017-10-05

**Authors:** Shwu-Maan Lee, Chia-Kuei Wu, Jordan L. Plieskatt, Kazutoyo Miura, John M. Hickey, C. Richter King

**Affiliations:** a PATH Malaria Vaccine Initiative (MVI), Washington, DC, USA; b Laboratory of Malaria and Vector Research, National Institute of Allergy and Infectious Diseases, National Institutes of Health, Rockville, Maryland, USA; c Macromolecule and Vaccine Stabilization Center, University of Kansas, Lawrence, Kansas, USA; CDC

**Keywords:** Pfs230, Plasmodium falciparum, baculovirus, malaria, recombinant protein, transmission-blocking vaccine

## Abstract

Transmission-blocking vaccines have the potential to accelerate malaria parasite elimination by inducing antibodies that block parasite transmission from humans to mosquitoes. Pfs230, a gametocyte surface protein involved in gamete function, has long been a promising candidate. Due to the large size (3,135 amino acids), complex domains, and repeating 6-cysteine (6-Cys) motifs with a multitude of disulfide bonds, the feasibility of expression of a full-length protein has been difficult. A priority focus, therefore, has been on the generation of single domains, including N-terminal fragments. Here we utilized a heterologous expression system, baculovirus, to produce an N-terminal domain of Pfs230 (Pfs230C1). Pfs230C1 (amino acids 443 to 731) with a polyhistidine affinity tag was expressed in Super Sf9 cells. Since the native host lacks glycosylation machinery, a single N585Q mutation was made to eliminate potential N-linked glycosylation. The expressed protein, purified by nickel affinity, ion exchange, and size exclusion chromatography to >90% purity, was present in monomeric form with an observed mass of 33,510 Da (matching oxidized form). Peptide mapping and disulfide analysis confirmed the proper formation of predicted disulfide bonds. Antibodies, generated against Pfs230C1 in mice, bound to the gametocyte in an immunofluorescence assay (IFA) and demonstrated functional activity in both the standard membrane feeding assay (SMFA) and the exflagellation assay (EXA). The biochemical, biophysical, and immunological results reported herein support the continued advancement of an N-terminal Pfs230 antigen (Pfs230C1) as a component of a transmission-blocking vaccine. Our results also support the continued use of the scalable baculovirus expression system for the generation of complex Plasmodium proteins.

## INTRODUCTION

Malaria caused by Plasmodium falciparum is responsible for nearly a half million deaths annually, on the basis of estimates from WHO (1), with most of those deaths occurring among young African children. The parasite has a complex life cycle, is highly adaptable, and has coevolved in humans for millennia. While the tools that save lives today, such as bed nets, insecticides, and drugs, have had a positive impact on the disease over the last decade, it is widely recognized that a vaccine capable of inducing immune responses that block the cycle of parasite transmission will accelerate elimination and eventual eradication efforts (2, 3).

The profile of vaccines needed to contribute to control, elimination, and eventual eradication efforts are outlined in the *Malaria Vaccine Technology Roadmap* (4). Transmission-blocking vaccines (TBVs) are designed to induce in human hosts antibodies against sexual-stage malaria antigens or mosquito antigens, thereby disrupting parasite development after an Anopheles mosquito takes a blood meal from a malaria parasite-infected individual. The small number of parasites present at this stage of the parasite life cycle (typically one to five) and the extended time needed prior to mosquito midgut traversal (∼24 h) present a major interventional opportunity. Successful targeting of this stage of the parasite life cycle via vaccine-induced antibodies would have a major impact on the probability that infectious sporozoites will be produced in the mosquito and, thus, transmitted to humans.

Pfs25, a protein expressed on the surface of zygotes and ookinetes, is the most advanced target for TBV candidates. Despite promising preclinical results, including in nonhuman primates, clinical studies have resulted in the poor induction of transmission-blocking antibodies (5, 6; see also the study registered at ClinicalTrials.gov under registration no. NCT02013687). Among other potential TBV targets, one of the most promising antigens is the Pfs230 protein, which is expressed on the surface of gametocytes and serves an essential role in gamete biology. Unlike Pf25, antibodies targeting Pfs230 and other antigens present on gametocytes, such as Pfs48/45, may be boosted by natural infection (7). A correlation between naturally acquired antibody responses against Pfs230 and Pfs48/45 and transmission-reducing activity in sera from patients in areas of endemicity was recently reviewed (8). Therefore, it may be advantageous to target both Pfs230 and Pfs48/45 in a TBV approach.

The Pfs230 protein has presented a formidable challenge for recombinant expression due to its large size (3,135 amino acids [aa]) and the large number of disulfide bonds present (9, 10). Carter et al. (11) predicted that Pfs230 has seven paired domains and that the transmission-blocking (TB) target epitopes are located within these motif-defined domains. Recombinant expression of full-length Pfs230 has not been reported, presumably due to its complexity. However, smaller fragments and those specifically in the N-terminal region have been produced and tested, indicating that Pfs230 induces transmission-blocking antibodies (12). Tachibana et al. (13) produced a recombinant Pfs230 domain from aa 443 to 1132 and three truncated forms of this Pfs230 N-terminal domain from aa 443 to 588, aa 443 to 715, and aa 443 to 915 in the wheat germ cell-free expression system (13). Furthermore, rabbit antibodies raised against these recombinant proteins displayed significant TB activity in the standard membrane feeding assay (SMFA) (13), indicating that fragments of Pfs230 may have vaccine potential. Recently, recombinant expression of N-terminal constructs of Pfs230 in other expression systems, including in plants (14), expressing aa 444 to 730, and in Pichia (15), expressing aa 542 to 736, has been reported.

A prudent consideration in the development of TBV antigens is identification of a suitable heterologous expression system able to produce soluble, properly folded recombinant proteins in sufficient quantity for development, optimization, and current GMP production. Previously, we demonstrated that properly folded Pfs25 can be expressed in the baculovirus expression system at reasonably high yields, with a high purity, and, most importantly, with demonstrated TB activity (16). In view of this success, we continued to explore the production of other leading TBV antigens in this system. Here, we report on the expression and the biochemical, biophysical, and immunological characterization of an N-terminal recombinant Pfs230 domain produced from the baculovirus system. We expressed a Pfs230 domain from aa 443 to 731, designated Pfs230C1, which is similar to the construct from aa 443 to 715 of Tachibana et al. (13) and the construct from aa 444 to 730 of Farrance et al. (14). Further, Pfs230C1 was shown to induce in mice antibodies that exhibited functional activity, as determined by an exflagellation assay (EXA) and SMFA.

The data presented here support further assessment of baculovirus-produced Pfs230C1 to support the development of an effective transmission-blocking vaccine. Our results also support the continued use of the scalable baculovirus expression system for the generation of complex malaria proteins.

## RESULTS

### Baculovirus produces Pfs230C1.

**FIG 1:**
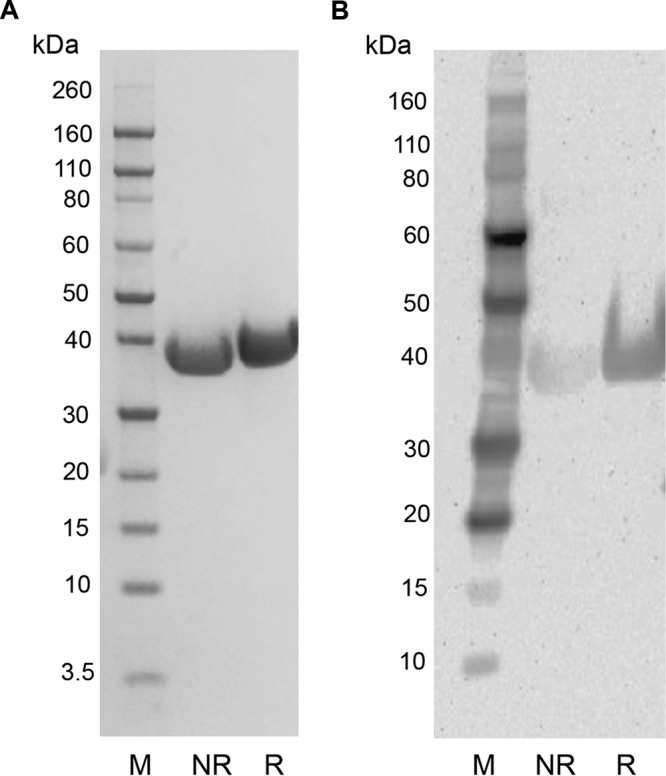
SDS-PAGE and Western blotting of Pfs230C1. The results of SDS-PAGE of Pfs230C1 under nonreducing conditions (lanes NR) and reducing conditions (lanes R) and staining with SimplyBlue SafeStain stain (A) and Western blotting with anti-His antibody (B) are shown. Lanes M, molecular mass markers.

We previously utilized the baculovirus expression system to produce TBV candidate antigens, including a high-quality, disulfide-rich Pfs25 (16). Taking into consideration a similar challenge from the multiple disulfides present in the Pfs230 antigen, we decided to extend the use of the scalable baculovirus system to the production of a soluble monomeric N-terminal domain of Pfs230 (Pfs230C1). The Pfs230C1 construct (aa 443 to 731) contained a signal peptide to facilitate expression of the protein through the endoplasmic reticulum secretion pathway, an N585Q mutation to remove a potential N-glycosylation site, and a 6-histidine C-terminal tag. We used a three-step purification approach to capture and polish Pfs230C1: Ni-nitrilotriacetic acid (NTA) affinity, DEAE ion exchange, and size exclusion (SE) chromatography. The initial process, as presented here, yielded 2 mg of purified Pfs230C1 per liter of culture supernatant. Further, the resulting purified protein was present at the expected molecular mass of ∼33.5 kDa and had greater than 90% purity by SDS-PAGE and densitometry (Fig. 1A). Reactivity to the anti-His antibody confirmed the presence of the histidine tag on the C terminus (Fig. 1B).

**FIG 2:**
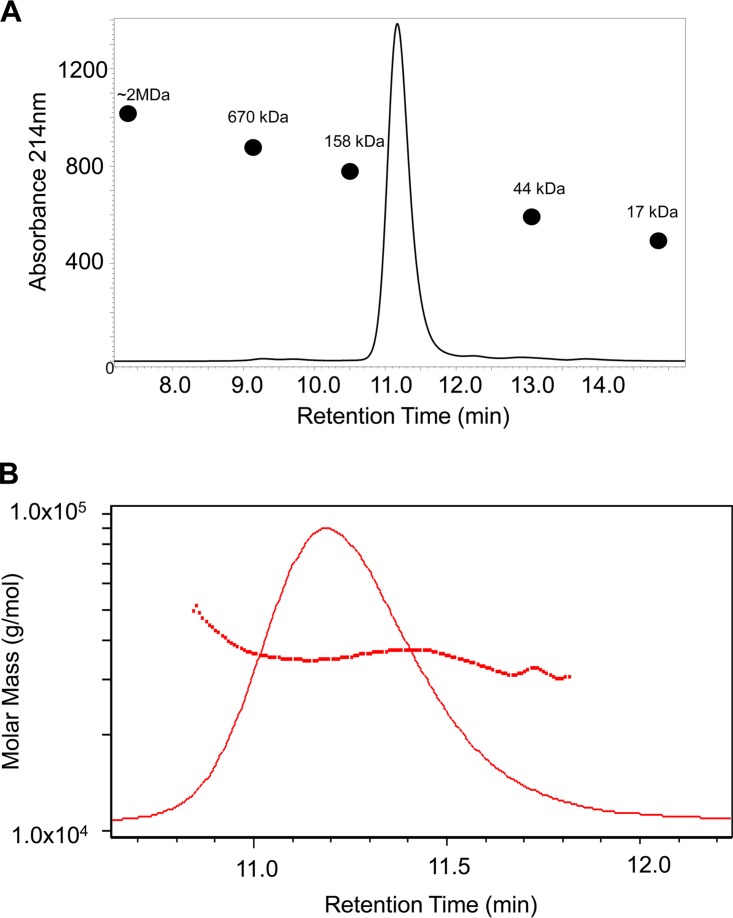
SE-HPLC (A) and multiangle light scattering showing the molar mass of Pfs230C1 (B). (A) The major peak of Pfs230C1 eluted between the 158-kDa and 44-kDa protein standards (indicated by black circles on the chromatogram) by SE-HPLC and was further assessed by multiangle light scattering. (B) The molar mass (red squares) determined by MALS plotted against the SE-HPLC elution peak (red line) indicated an average molar mass of 35.2 ± 0.5 kDa, consistent with the molecular mass of a Pfs230C1 monomer.

Analytical size exclusion chromatography was performed to assess the solution state of the purified recombinant protein. Pfs230C1 eluted between the 158- and 44-kDa standards, which was larger than the expected size of the Pfs230C1 monomer, which is ∼33.5 kDa (Fig. 2A). Given that Pfs230C1 eluted at a mass greater than the 44-kDa standard and possibly existed in a higher oligomer state, we utilized multiangle light scattering (MALS) to confirm the molar mass in solution (Fig. 2B). The average molar mass of Pfs230C1 from MALS analysis was 35.2 ± 0.5 kDa, consistent with the molecular mass of a monomer (∼33.5 kDa, confirmed by intact mass analysis). This indicated that the short retention time was most likely attributed to an expanded hydrodynamic radius relative to that of the size exclusion chromatography protein standards.

### Formation of disulfide bonds.

Pfs230C1 contains a predicted cysteine-rich domain, with four cysteines forming two disulfide bonds (10). The cysteine-rich domains are well characterized and present in protein families, such as Pfs230 and Pfs48/45, both of which play an important role in the fertility of gametes (17–19) in sexual stages. Proper disulfide bond formation is important to maintain native epitopes and ensure induction of functional antibodies. To determine if the cysteines were free or formed disulfide bonds within the protein, the number of thiol groups exposed on the protein surface was measured. The analysis showed that Pfs230C1 contained little (<1%) or no free thiol under both native and denatured conditions (3 M guanidine-HCl [GuHCl]), suggesting that all four cysteines were fully oxidized and likely paired by disulfide bonds.

### Glycosylation of Pfs230C1 was limited.

**FIG 3:**
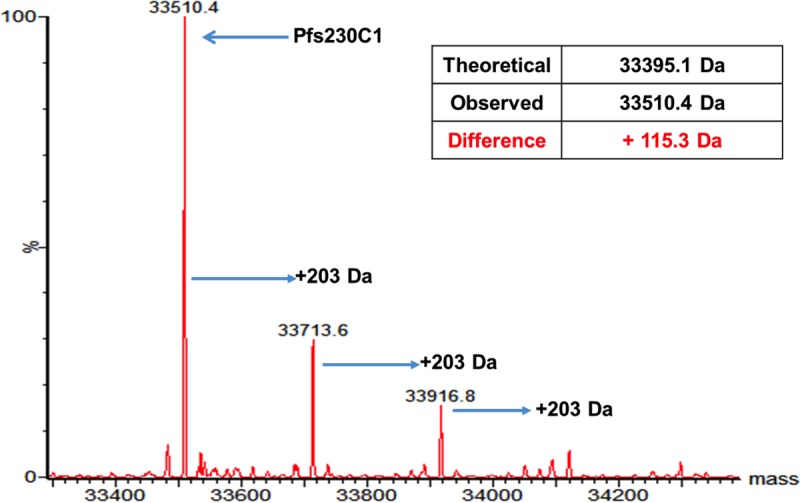
Intact mass spectrometry of Pfs230C1 under nonreducing conditions. Arrows, predominant mass of 33,510 and adducts with masses greater than the predominant mass in 203-Da increments most likely resulting from *O*-linked glycosylation.

Proteins on parasite surfaces are, in general, not glycosylated. To determine whether the baculovirus-expressed recombinant Pfs230C1 protein was subject to glycosylation or other posttranslational modifications, we performed intact mass and peptide mapping analyses. The results indicated that the observed mass of the Pfs230C1 was +115 Da higher (at 33,510 Da) than its theoretical value of 33,395 Da (Fig. 3). Since the protein was expressed via the secretion pathway in the insect cells, the mass difference was likely due to the inclusion of an N-terminal Asp residue of the signal peptide; this finding is supported by a similar result reported previously (16). Using liquid chromatography (LC)-mass spectrometry (MS) and peptide mapping, the N-terminal amino acid was confirmed to be aspartic acid. After correction for the Asp addition at the N terminus, the predominant intact mass of 33,510.4 Da matched the predicted mass for the oxidized form of the Pfs230 fragment (Fig. 3), confirming the formation of disulfide bonds.

Minor species with increases in mass from the predominant intact mass (33,510 Da) in 203-Da increments were observed, and these were potentially due to the presence of mono- or oligosaccharides (Fig. 3). High-resolution peptide mapping by MS/MS located one of the glycosylation sites, Ser^110^, in peptide (101)-SVLQSGALPSVGVDELDK-(118), which was most likely attributed to *N*-acetylglucosamine or *N*-acetylgalactosamine, an *O*-linked glycosylation. We interpret these results to indicate that the overall glycosylation modification on Pfs230C1 is minor, with the dominant purified species being unmodified.

### Disulfide mapping.

**FIG 4:**
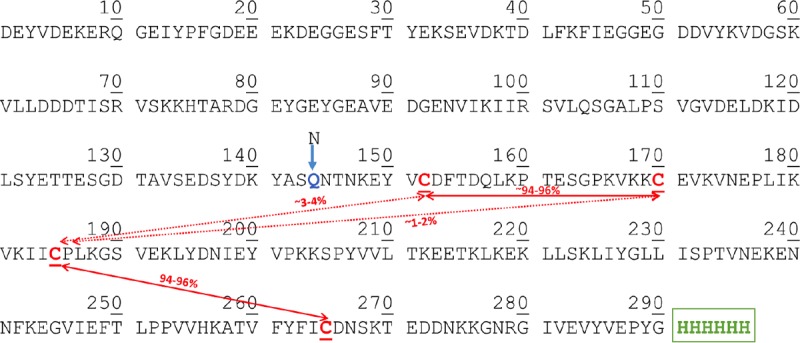
Disulfide analysis of Pfs230C1. Pfs230C1 containing four cysteine residues is expected to form two disulfide bonds. Using peptides from nonreduced trypsin and chymotrypsin digestion, >95% of the disulfide bonds were properly formed. Boxes indicate Cys 1-Cys 2 and Cys 3-Cys 4 disulfide bond formations with approximately 1 to 2% misformed Cys 2-Cys3 bonds and 3 to 4% misformed Cys 1-Cys 3 bonds.

Given the importance of proper disulfide bond formation for the native configuration of Plasmodium proteins, we asked whether the cysteines of recombinant Pfs230C1 were correctly paired. The expressed protein contained one predicted cysteine-rich motif with an expected pattern for the formation of the disulfide bonds. Peptide mapping was performed on a nonreduced sample of Pfs230C1, with the resulting sequence coverage of the trypsin- and chymotrypsin-digested forms (Table 1) being 86% and 88%, respectively. The combined sequence coverage was 96%, with many peptides overlapping. The LC-MS characteristics of each identified disulfide-linked peptide are reported in Table 1. The relative percentages of the disulfide bonds were calculated using the ion intensity (Table 1) of each disulfide-linked peptide containing each Cys residue. The results indicated that four Cys residues (Cys^152^, Cys^170^, Cys^185^, and Cys^265^) in Pfs230C1 formed two disulfide bonds, between Cys^152^ and Cys^170^ and between Cys^185^ and Cys^265^ (Fig. 4; Table 1). The pairing of the Pfs230C1 cysteines confirmed the prediction of the disulfide bond pattern of the cysteine-rich motif (10). Less than 5% mispaired disulfides were observed (Fig. 4). We further interpreted these data to indicate that the expressed Pfs230C1 protein adopts a single, dominant configuration and that the disulfide bonding pattern most likely mimics that of the native full-length equivalent.

**TABLE 1:** Characteristics of LC-MS analysis of disulfide-linked peptides from nonreduced Pfs230C1

Protease	Retention time (min)	Monoisotopic *m/z*	MS area	Charge	Observed monoisotopic mass (Da)	Theoretical monoisotopic mass (Da)	Mass difference^*a*^	Disulfide-linked peptides
Trypsin	28.8	963.2	3.3E+04	3	2,886.6	2,886.4	0.2	V167-K173/E149-K166
Trypsin	29.7	887.4	1.0E+05	3	2,659.2	2,659.2	0	K169-K173/E149-K166
Trypsin	30.8	1,266.6	9.4E+04	2	2,531.2	2,531.2	0	C170-K173/E149-K166
Trypsin	30.8	844.8	1.7E+05	3	2,531.4	2,531.2	0.2	C170-K173/E149-K166
Trypsin	26.4	581.3	8.4E+03	2	1,160.6	1,160.7	−0.1	C170-K173/I183-K188
Trypsin	46.8	1,046.1	2.1E+05	2	2,090.2	2,090.1	0.1	I183-K188/A258-K269
Trypsin	39.8	914.2	3.3E+04	3	2,739.6	2,739.4	0.2	I183-K188/E149-K166
Trypsin	46.8	697.8	2.0E+05	3	2,090.4	2,090.1	0.3	I183-K188/A258-K269
Chymotrypsin	35.0	1,049.9	2.3E+04	3	3,146.7	3,146.6	0.1	V151-F154/T155-L178
Chymotrypsin	35.2	1,049.9	1.8E+04	3	3,146.7	3,146.6	0.1	V151-F154/T155-L178
Chymotrypsin	36.2	1,049.9	1.5E+05	3	3,146.7	3,146.6	0.1	V151-L158/K159-L178
Chymotrypsin	35.2	1,049.9	7.3E+04	3	3,146.7	3,146.6	0.1	V151-L158/K159-L178

a The mass difference was calculated as the observed mass minus the theoretical mass.

### Baculovirus-expressed Pfs230C1 elicits functional antibodies.

**FIG 5:**
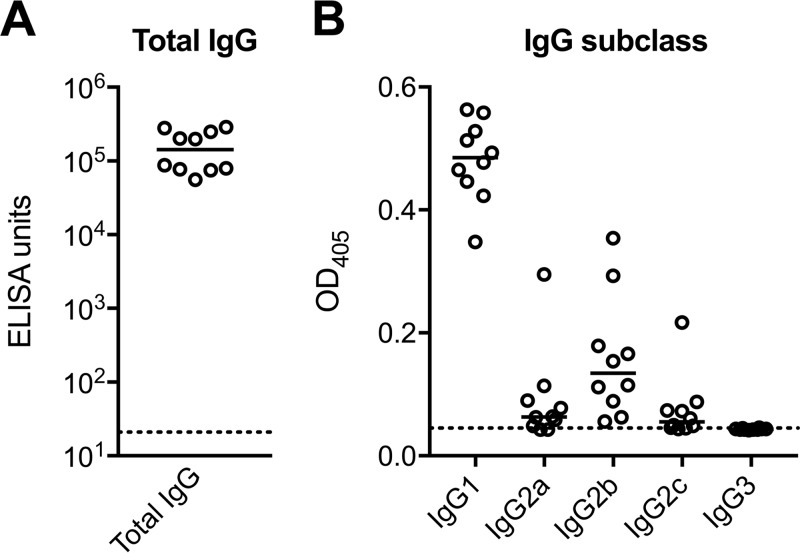
ELISA titers in mice immunized with Pfs230C1. ELISA data for individual mice (circles) and medians (bar) are shown. Total anti-Pfs230C1 antibody levels are presented on a log scale of ELISA units (A), and IgG subclass results are given as the optical density at 405 nm (OD_405_) (B). Dotted lines indicate the background signals.

**FIG 6:**
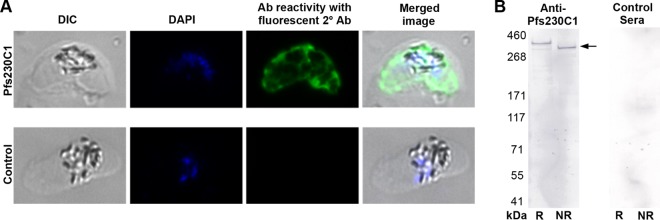
Immunofluorescence assay (IFA) and Western blotting (WB). (A) An immunofluorescence assay was utilized to confirm the expression and localization of Pfs230 (or Pfs230C1) in fixed mature gametocytes. Primary antibodies of anti-Pfs230C1 (top) or antiadjuvant negative-control (bottom) purified IgGs were incubated with fixed parasites. The results of differential interference contrast (DIC) microscopy, DNA staining with DAPI (4′,6-diamidino-2-phenylindole), and antibody (Ab) reactivity to the parasite with fluorescence-labeled anti-mouse IgG secondary antibody and a merged image of gametocytes are shown. (B) Western blot using anti-Pfs230C1 (left) or control (right) antisera. Lanes R, reducing conditions; lanes NR, nonreducing conditions. The Pfs230 band (expected molecular mass, 363 kDa) is indicated by the arrow.

**FIG 7:**
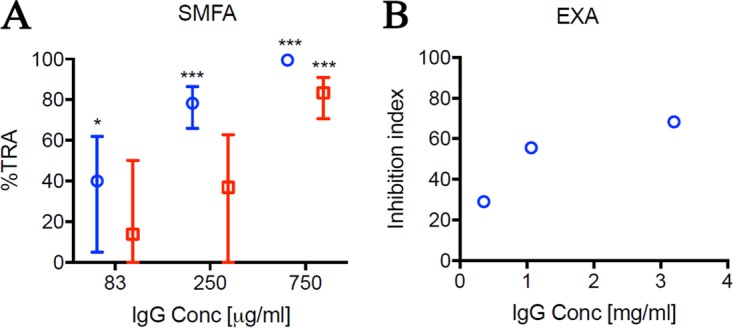
Functional activity of anti-Pfs230C1 antibody. (A) The anti-Pfs230C1 antibody was tested by SMFA at 83, 250, and 750 μg/ml in the presence (three independent assays; blue) or absence (two independent assays; red) of complement. The best estimate and 95% confidence intervals of the percent TRA from the multiple feeding experiments are shown. The asterisks indicate whether the levels of inhibition were significantly different from no inhibition (i.e., 0% inhibition). *, *P* < 0.05; ***, *P* < 0.0001. (B) The same antibody was tested by EXA at 0.4, 1.1, and 3.2 mg/ml in the presence of complement. The inhibition index was calculated determination of inhibition against that for the antiadjuvant antibody tested in the same assay.

To further confirm that the expressed protein indeed represented the native conformation, a mouse immunogenicity study was conducted to determine whether the antibodies elicited by Pfs230C1 bound native protein on the parasite and exhibited functional activity against parasite development. Individual serum samples from mice immunized with 16.5 μg Pfs230C1 adjuvanted with Montanide ISA720 or Montanide ISA720 alone (negative control) were analyzed by enzyme-linked immunosorbent assay (ELISA) for total IgG (Fig. 5A) and further for the IgG subclass (Fig. 5B). Mice immunized with Pfs230C1 had a mean total IgG titer of greater than 100,000 ELISA units, and the antibodies were predominantly of the IgG1 subclass, followed by the IgG2b subclass (Fig. 5). Serum samples were then pooled for each group, and total IgGs were purified for analysis by immunofluorescence assay (IFA) (Fig. 6A). The IFA results indicated that the purified IgG from Pfs230C1 antiserum recognized the surface of stage V gametocytes, where Pfs230 is localized (Fig. 6A). Antisera from mice immunized with Pfs230C1 reacted with a fixed mature gametocyte extract via Western blot analysis under both reducing and nonreducing conditions, including positive reactivity at approximately 363 kDa, the expected molecular mass of full-length Pfs230 (Fig. 6B). Next the functional activities of the anti-Pfs230C1 antibody were evaluated by SMFA and EXA (Fig. 7). Purified IgG from Pfs230C1 antiserum, tested at 750 μg/ml in the SMFA (with complement), was associated with a 99.5% reduction (95% confidence interval [CI], 98.3% to 100%; *P* < 0.0001) in oocyst density. To determine the dose dependency of inhibition, purified IgG was tested at 3-fold dilutions in two (without complement) and three (with complement) independent assays (Fig. 7A). A dose-dependent reduction in oocyst intensity was observed; however, the levels of inhibition were weaker in the absence of complement. The data suggest that complement plays an important role, but is not required, for the transmission-reducing activity of Pfs230C1-induced antibodies. These data, generated from studies with mice, are generally consistent with those from previous reports for a similar Pfs230 fragment tested in rabbits (13). Lastly, in EXA, the antibody showed a dose-dependent reduction in the numbers of exflagellation centers (Fig. 7B). In contrast to the Pfs230 knockout parasites published by Eksi et al. (20), we observed a reduction of exflagellation and not the blocking of red blood cell (RBC) clustering in the EXA with anti-Pfs230 antibody.

In summary, the IFA, SMFA, and EXA results indicate that immunization with Pfs230C1, expressed in the baculovirus system, elicits antibodies that effectively target the parasite and inhibit its development and that are therefore expected to interrupt parasite transmission.

## DISCUSSION

The results reported herein confirm and extend the findings of previous work exploring the potential of Pfs230 protein fragments to support the rational design of a malaria transmission-blocking vaccine. Similar to others, we focused on an N-terminal fragment of Pfs230 to overcome the production challenges associated with a 350-aa disulfide-rich protein. The fragment that we selected and the expression system that we used are different from those reported by MacDonald et al. (15). They reported on the characterization of a Pfs230 construct (encoding aa 444 to 736) expressed by Pichia pastoris and observed consistent cleavage at position 542. To circumvent the cleavage issue, they restricted their construct to aa 542 to 736. In the study reported here, we did not observe proteolytic processing at aa 542 when expressing Pfs230C1 (aa 443 to 731) in baculovirus. This could be due to less protease activity in the baculovirus system than in the Pichia system.

Our work also confirms domain predictions regarding the multiple cysteine-rich (6-cysteine) domains within Pfs230. Carter et al. (11) first proposed that Pfs230 is composed of multiple 6-Cys domains with a predicted and conserved set of disulfide bonds in the following pairing pattern: Cys 1 and 2, Cys 3 and 6, and Cys 4 and 5. These 6-Cys domains form a beta-sandwich with a mixture of parallel and antiparallel strands (17, 21). More specifically, the Cys 1-Cys 2 and Cys 3-Cys 6 pairs pin together the two beta-sheets of the sandwich, whereas the Cys 4-Cys 5 pair links an ancillary loop to the core domain and is not always required or conserved (10, 17). Several 6-Cys proteins also contain one or more such 4-cysteine folds (22), similar to the Pfs230C1 studied here. However, the term “6-Cys domain” has been utilized by others while still referring to 4-cysteine folds (15). To avoid confusion and maintain convention, we chose to apply the 6-Cys nomenclature to Pfs230C1 throughout this article, even though only four cysteines are present.

**FIG 8:**
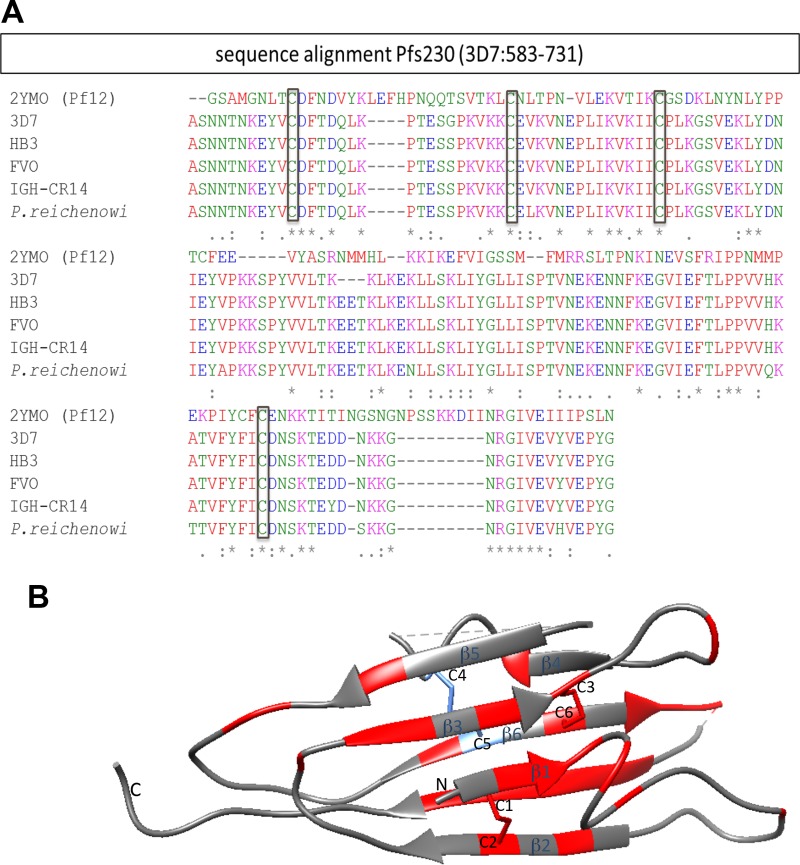
Alignment of the 6-Cys domain sequence within Pfs230C1 with the sequence of Pf12 (A) and ribbon diagram (B). (A) Pfs230 sequences (aa 583 to 731) from different strains and a closely related species of Plasmodium were used to align with the A-type 6-Cys fold of the Pf12 crystal structure (PDB accession number 2YMO, aa 1 to 173). These included the sequences of Plasmodium falciparum strains 3D7 (GenBank accession number P68874, aa 583 to 731), HB3 (GenBank accession number CH671929.1), FVO (GenBank accession number KI925011), and IGH-GR14 (GG665179) and Plasmodium reichenowi (GenBank accession number LVLA01000003). Asterisks, conserved cysteines and other residues; colons, strongly similar residues; periods, weakly similar residues (31). The physicochemical properties of each residue are denoted by color, where red, blue, magenta, and green indicate hydrophobic, acidic, basic, and hydroxyl/sulfhydryl/amine residues, respectively. (B) Ribbon diagram of the conserved residues among Pfs230 strains and Pf12 modeled using Chimera software (32) on the basis of the crystal structure of Pf12 (PDB accession number 2YMO). The conserved sequences (red) resided across the structure but resided more densely on beta-sheets, and the four cysteines (Cys 1, Cys 2, Cys 3, and Cys 6) forming two disulfide bonds can be visualized.

Pf12, a merozoite surface protein, is the only 6-Cys protein for which the high-resolution structure has been solved (17, 21); therefore, we compared the 6-Cys domain within Pfs230C1 to that in Pf12 by (i) sequence alignment and (ii) mapping of the conserved amino acid residue on the Pf12 structure. The 6-Cys fold of Pfs230C1 (aa 583 to 731) lies within the first of seven double domains in Pfs230 (10). In addition to Plasmodium falciparum 3D7, sequences covering 6-Cys domains from different strains and a closely related species of Plasmodium were also used to align with the sequence covering the 6-Cys fold of the Pf12 crystal structure (Protein Date Bank [PDB] accession number 2YMO, aa 1 to 173). This alignment of cysteine residues (Fig. 8A) suggests that disulfide bond formation would generate a similar conformation for Pf12 and Pfs230C1. Our biochemical characterization confirmed these predicted disulfide bonds in Pfs230C1. Based on this finding, we mapped the Pfs230C1 sequence onto the Pf12 structure, with conserved residues primarily being identified within the characteristic beta-sheets (Fig. 8B).

Our analysis also helps explain the biochemical properties of the Pfs230C1 protein. While initial size analysis of Pfs230C1, using SE high-performance liquid chromatography (SE-HPLC), indicated that Pfs230C1 eluted between the 158- and 44-kDa protein standards, multiangle light scattering (MALS) showed that the average molar mass of Pfs230C1 was 35.2 ± 0.5 kDa. These data suggest that purified Pfs230C1 is a monomer and has a relatively large hydrodynamic radius, likely attributed to the barrel shape predicted above (Fig. 8B) and the N-terminal nonstructural region of Pfs230C1 (10).

In addition to confirmation of the presence of disulfide bonds, the proper conformation of Pfs230C1 was suggested by the elicitation of functional antibodies measured by two orthogonal assays. First, we showed clear evidence of the functional activity of anti-Pfs230C1 serum in the SMFA. In this well-characterized and frequently used assay, antibodies are ingested by Anopheline mosquitoes along with parasites in the blood meal (23). The subsequent ability of antibodies to cause a reduction in the development of oocysts can be readily determined by microscopic examination. The Pfs230C1 fragment reported here elicited serum samples that readily reduced the oocyst density in this assay, and moreover, the sera did not completely depend on complement for activity, as has been reported for many antibody preparations reactive to Pfs230 (13). Second, we saw clear evidence that anti-Pfs230C1 antibodies exhibited functional activity, as measured by inhibition in the formation of exflagellation centers. Eksi et al. (24) disrupted the Pfs230 gene in two different N-terminal locations (aa 452 and aa 950), which resulted in the production of only the N-terminal region of the protein and, consequently, mislocated and truncated Pfs230 proteins not directly associated with the gametocyte and the gamete surface. While the Pfs230 disruptants successfully emerged from RBCs and the male gametes exflagellated, producing microgametes, the exflagellating Pfs230-negative males were unable to form exflagellation centers (20). Our result supports these functional assessments of Pfs230, as the purified IgG elicited by the N-terminal domain protein (Pfs230C1) described here was also able to inhibit the formation of exflagellation centers.

The ability to elicit functional transmission-blocking antibodies is the most important characteristic of any protein preparation to be considered for the generation of a TBV. Given that P. falciparum apparently lacks glycosylation modifications, it has been a concern that the preparation of recombinant proteins in eukaryotic systems could result in proteins that are suboptimal in their capacity to induce highly effective functional antibody responses. In this study, the limited *O*-linked glycosylation detected in recombinant Pfs230C1 did not appear to affect the ability to elicit potent transmission-blocking antibodies, as tested by SMFA. Lastly, the vaccine formulation used in this study induced a predominantly IgG1 response, followed by an IgG2b response. IgG1 is known to have very low or no complement-fixing activity (25). Therefore, it is possible that the IgG2b-induced activity was affected by the presence (or absence) of complement, but this was not the case for IgG1-induced activity. Further study is required to uncover the mechanism.

In summary, our results indicate that Pfs230C1, expressed in the baculovirus system, harbors many of the critical biochemical and immunological attributes required to support consideration of its use in a TBV approach. While initial experimentation provided a final purified protein yield of 2 mg Pfs230C1 per liter culture supernatant, further optimization of the process improved this yield to over 10 mg/liter with optimization of upstream processes, including optimization of the cell density, the culture medium, and the multiplicity of infection (MOI). Given the scalability of the system (26) and access to sufficient bioreactor volumes, this current yield would provide sufficient material for further vaccine development and later clinical evaluation. Further, there are now at least six approved vaccines based on the baculovirus system, including four for use in humans (27), increasing interest in and regulatory support for products derived from this expression system.

We intend to continue the process of development of Pfs230C1 and explore the incorporation of the Pfs230C1 protein into different vaccine delivery platforms and adjuvant combinations for the generation of malaria TBV candidates.

## MATERIALS AND METHODS

### Baculovirus expression construct (Pfs230C1).

The N-terminal sequence (aa 443 to 731) of the gametocyte surface protein Pfs230 of the P. falciparum 3D7 strain (GenBank accession number P68874) containing four cysteines as part of a predicted cysteine-rich domain was cloned and is denoted Pfs230C1.

Codon optimization for baculovirus expression was performed by DNA2.0. Synthetic DNA of Pfs230C1 (aa 443 to 731) containing the N585Q mutation to remove a potential N-glycosylation site and containing an additional N-terminal secretion signal (MKFLVNVALVFMVVYISYIYAD from honeybee melittin) was cloned into the pFastBac donor vector (Invitrogen) with BamHI (5′) and EcoRI (3′) sites. The sequence of the resulting plasmid, pFastBac-Pfs230C1, was verified. The generation of recombinant viruses was done by following the instructions of the manufacturer of the Bac-to-Bac system (Invitrogen). Briefly, pFastBac-Pfs230C1 was transformed into Escherichia coli DH10Bac; colonies were grown at 37°C for 48 h on LB agar plates containing tetracycline (10 μg/ml), kanamycin (50 μg/ml), gentamicin (7 μg/ml), IPTG (isopropyl-β-d-thiogalactopyranoside; 40 μg/ml), and X-Gal (5-bromo-4-chloro-3-indolyl-β-d-galactopyranoside; 100 μg/ml). The sequence of the recombinant bacmid was confirmed by PCR and sequencing. This bacmid was used to transfect Super Sf9 cells (Oxford Expression Technologies) for the generation of a recombinant baculovirus stock (passage 1 [P1] virus), using the Cellfectin II reagent (Invitrogen) following the instructions in the Bac-to-Bac manual. P1 virus was harvested at 96 h posttransfection and stored at 4°C with protection from light. Two milliliters out of the 14 ml of P1 virus harvested was used to amplify P2 baculovirus after infecting fresh Super Sf9 cells at 27°C for approximately 96 h and, similarly, to produce high-titer P3 viruses. A P3 virus volume of 400 ml for further expression was harvested, and the titer was determined using a BacPAK titer determination kit (Clontech).

### Expression and purification of Pfs230C1.

Super Sf9 cells were seeded at 1 × 10^6^ cells/ml in ESF 921 medium (Expression Systems). An MOI of 1 was used to infect a 10-liter Super Sf9 cell wave culture. At 48 h postinfection, the culture was harvested and concentrated using a 10-kDa tangential flow filtration membrane (0.1 m^2^; Millipore) and diafiltered with 20 mM sodium phosphate, 150 mM NaCl, pH 7.4 (buffer A). Clarification was carried out by filtration through a 0.22-μm-pore-size filter.

Two liters of clarified supernatant was batch bound to 10 ml Ni-NTA resin (His60; Clontech) overnight. The wash steps were performed with 5 column volumes (CV) of buffer A with 10 mM imidazole and then 5 CV of buffer A with 20 mM imidazole and 5 CV of buffer A containing 30 mM imidazole. The protein was eluted with buffer A containing 300 mM imidazole. Pooled eluents from the Ni-NTA column were diluted 8-fold with 20 mM sodium phosphate buffer, pH 7.2, containing 5% glycerol and loaded onto a DEAE-Sepharose FF column with 10 ml resin for ion exchange purification. The column was washed with 5 CV of 20 mM sodium phosphate, 20 mM NaCl, 5% glycerol, pH 7.2 (buffer B), and eluted with an NaCl gradient from 0.02 to 1 M in 10 CV in buffer B. The pool of eluents was concentrated to 15 ml and loaded onto a Superdex 200 (16/60; GE Healthcare) column for a final step of size exclusion purification and buffer exchange into 20 mM sodium phosphate, 150 mM NaCl containing 5% glycerol (pH 7.2). The eluents were collected and analyzed by SDS-PAGE and Western blotting, and a final pool was selected on the basis of purity.

### SDS-PAGE.

Samples were diluted with 4× lithium dodecyl sulfate (LDS; Invitrogen) sample buffer, heated for 10 min, and loaded in a final volume of 20 μl/well onto SDS-polyacrylamide gels (4 to 12% NuPAGE bis-Tris gels; Invitrogen). The gels were run at 150 to 200 V for 35 to 50 min in 1× MES (morpholineethanesulfonic acid)-SDS running buffer and stained with SimplyBlue SafeStain stain (Invitrogen).

### Western blotting with purified Pfs230C1.

Following SDS-PAGE, the proteins were transferred onto a nitrocellulose membrane and blocked in 5% skim milk in PBS at room temperature for 1 h. Primary antibody (Penta His antibody; Qiagen) at a 1:2,000 dilution in 1% skim milk in phosphate-buffered saline (PBS) containing 0.05% Tween 20 (PBST) was added, and the mixture was incubated for 2 h at room temperature. The membranes were washed with PBST (3 times for 5 min each time), secondary antibody consisting of a 1:5,000 dilution of goat anti-mouse IgG–horseradish peroxidase (Santa Cruz) in 1% skim milk (PBST) was added, and the mixture was incubated at room temperature for 1 h. The membranes were then again washed with PBST (3 times for 5 min each time) and developed using 3,3,5,5′-tetramethylbenzidine (TMB; Sigma-Aldrich) and hydrogen peroxide.

### Kinetic endotoxin assay.

A SpectraMax Plus spectrophotometer (kinetic endotoxin assay) was used to quantify the endotoxin content of purified Pfs230C1 with EndoSafe endotoxin (Charles River), EndoSafe lysate (Charles River), and EndoSafe Limulus amoebocyte lysate reagent water (Charles River).

### SE-HPLC.

SE-HPLC analysis of purified Pfs230C1 was performed on a BioAssist G3SWxl column (7.8 by 300 mm; Tosoh Biosciences, King of Prussia, PA) at 30°C on a Shimadzu Prominance ultrafast liquid chromatography HPLC system. The mobile phase consisted of 0.2 M sodium phosphate, pH 6.8, with flow rate of 0.7 ml/min. A gel filtration standard (Bio-Rad, Hercules, CA) was used to ensure column and HPLC system integrity. Detection by multiangle light scattering (MALS) was conducted using a Dawn Heleos and Optilab rEX instrument (Wyatt Technologies) connected directly after the HPLC system. A differential index of refraction (*dn*/*dc*) value of 0.185 ml/g (theoretical value) was used to calculate the protein's molar mass.

### Free thiol determination.

The amount of free thiol (the number of free cysteine residues) was measured using a Measure iT free thiol assay kit (Life Technologies, Carlsbad, CA) following the manufacturer's instructions. Pfs230C1 was diluted (in triplicate) in either ultrapure water or 3 M guanidine-HCl to achieve a final concentration of 30 μM before the assay was conducted. A standard curve was constructed using known concentrations of reduced glutathione. Fluorescence was measured using a SpectraMax M5 plate reader (Molecular Devices, Sunnyvale, CA).

### Intact mass spectrometry.

Pfs230C1 was denatured with 3 M GuHCl and desalted on a reversed-phase PRP-1 column (1 cm by 1 mm [inside diameter]; particles [10 μm] were packed by hand; Hamilton) using a NanoAcquity chromatographic system (Waters Corporation) and solvents A (99.9% H_2_O, 0.1% formic acid) and B (99.9% acetonitrile, 0.1% formic acid) over a short gradient from 1 to 70% solvent B in 4 min at a flow rate of 20 μl/min. The intact mass of the protein (in duplicate experiments) was measured using a Synapt G2 hybrid quadrupole/ion mobility/time of flight mass spectrometer (Waters Corporation, Milford, MA) with the assistance of the Mass Spectrometry and Analytical Proteomics Laboratory at the University of Kansas. The instrument was operated in a sensitivity mode with all lenses optimized on the MH^+^ ion from the control leucine enkephalin and a sample cone voltage of 40 eV. Argon was admitted to the trap cell operated at 4 eV for maximum transmission. Spectra were acquired at a 9,091-Hz pusher frequency covering a mass range of 100 to 3,000 *m/z*, and data were accumulated for 2 s per cycle. The time to mass calibration was made with NaI cluster ions acquired under the same conditions. The mass spectra of [Glu^1^]fibrinopeptide B were acquired in parallel scans, and doubly charged ions at *m/z* 785.8426 were used as a lock mass reference. MassLynx (version 4.1) software (Waters Corporation) was used to collect data and deconvolute the protein spectra for molecular mass determination.

### LC-MS peptide mapping for disulfide bond analysis.

Nonreduced Pfs230C1 was digested overnight at 37°C with either trypsin or chymotrypsin. The digested peptides were then subjected to LC-MS using a C_18_ column (150 by 2.1 mm; particle size, 1.7 μm; Thermo Fisher Scientific, Waltham, MA) operated at 60°C on an UltiMate 3000 ultra-high-performance liquid chromatography system (Thermo Fisher Scientific). The mobile phases consisted of A (water plus 0.05% trifluoroacetic acid [TFA]) and B (acetonitrile plus 0.05% TFA), and the peptides were eluted using a 0 to 70% gradient of mobile phase B over 60 min and a 0.2-ml/min flow rate. Peptides were identified using an LTQ-XL ion-trap mass spectrometer (Therμo Fisher Scientific) in the positive ion mode and a mass range of 300 to 2,000 *m/z*. Data files were processed using PepFinder (version 2.0) software (Thermo Fisher Scientific), and the search database consisted of the primary sequences of Pfs230C1, trypsin, and chymotrypsin. The peptide assignments of the MS/MS spectra were validated using a confidence score of ≥95% and some manual validation. The percentage of disulfide pairs was quantified by calculating the ion abundance of each disulfide-linked peptide and then calculating the relative abundance of each Cys residue within the different pairs of peptides.

### Mouse immunization.

To produce antisera in CD-1 mice for SMFA, 16.5 μg of Pfs230C1 was formulated with the Montanide ISA720 adjuvant. Groups of 10 mice (treated with Pfs230C1 and the adjuvant control) received intramuscular injections on day 0 and 21. It was confirmed, prior to immunization, that the purified protein contained less than 2 endotoxin units/mg endotoxin. On day 42, serum samples were collected, serum samples from individual animals in each group were pooled, and total IgG was affinity purified using a protein G column.

### Ethics statement.

The animal study reported here was conducted at the Laboratory of Malaria and Vector Research (LMVR) of NIAID, NIH, in compliance with Animal Welfare Act regulations and the guidelines in the *Guide for the Care and Use of Laboratory Animals* (28) and was reviewed and approved by NIAID's Animal Care and Use Committee (approval number LMVR10E).

### ELISA.

The basic methodology of the regular (total IgG) ELISA has been described elsewhere (29). The IgG subclass ELISA was performed with small modifications; all serum samples were diluted to 1 total IgG ELISA unit and then tested with anti-mouse IgG subclass-specific secondary antibodies (Southern Biotech or Bio-Rad). The results of the IgG subclass ELISA are presented using straight optical density (OD) values instead of the ELISA units used for the total IgG ELISA.

### Western blotting with parasite extract and mouse anti-Pfs230C1 antisera.

Two pools of mouse serum samples (one pool from the group receiving Pfs230C1 and another pool from the control group) were generated and tested at a 1:4,000 dilution against a parasite extract (5 × 10^4^ parasites per lane) by Western blotting. The mixture of P. falciparum NF54 gametocyte-, zygote-, and ookinete-stage parasites was made as described previously (16).

### Immunofluorescence assay (IFA).

Thin smears with mature gametocytes were made on slide glasses and air dried. The cells were then fixed and permeabilized with a 1:1 mixture of methanol-acetone at 4°C for 10 min. The slides were blocked at 37°C for 30 min with 1× PBS with 3% skim milk. Primary antibodies (either anti-Pfs230C1 or anti-adjuvant purified IgG at 1 μg/ml) were incubated at 37°C for 1 h. After three washes with 1× PBS, the slide was incubated with secondary antibody (Alexa Fluor 488-conjugated goat anti-mouse immunoglobulin antibody, 1:4,000 dilution) at 37°C for 30 min. The slide was washed again, DNA was stained with DAPI (4′,6-diamidino-2-phenylindole; ProLong Gold antifade reagent with DAPI) overnight at room temperature, and then the slide was examined under a Leica CTR6000 microscope.

### Antibody functional analysis (SMFA and EXA).

The anti-Pfs230C1 IgG was tested by SMFA at 750, 250, and 83 μg/ml with or without complement in two to four independent assays. In each assay, the anti-adjuvant IgG was tested at 750 μg/ml as a negative control. The standardized methodology for performing SMFA has been described previously (23). Briefly, 16- to 18-day-old gametocyte cultures of the P. falciparum NF54 line were mixed with a control or test sample and fed to ∼50 female Anopheles stephensi mosquitoes through a membrane-feeding apparatus. Eight days after the feed, the mosquitoes (*n* = 20) were dissected to enumerate the oocysts in the midgut. Only midguts from mosquitoes with any eggs at the time of dissection were analyzed.

The percent inhibition of the mean oocyst intensity (percent transmission-reducing activity [TRA]) was calculated as 100 × [1 − (mean number of oocysts in the anti-Pfs230C1 group)/(mean number of oocysts in the control group)]. Percent inhibition for a test sample was always calculated against that for a control sample examined in the same feeding experiment. The best estimate and 95% confidence intervals (CIs) of the percent TRA and the *P* value (for whether the percent inhibition was significantly different from no inhibition, i.e., 0% inhibition) from the multiple feeding experiments for each test condition at each concentration were calculated using a negative binomial model with zero inflation, as described previously (30).

Using the mature gametocyte cultures, EXA was performed by mixing 7.5 μl of packed red blood cells (0.5 to 1.0% stage V gametocytemia), 22.5 μl of non-heat-inactivated normal human serum, and 20 μl of antibody sample in 1× PBS. The mixture was incubated at 19°C for 18 min and then loaded onto a cellometer to determine the number of exflagellation centers. The inhibition index was calculated by comparing the number of exflagellation centers obtained with a test antibody with the number obtained with a control antibody tested in the same assay. An inhibition index of 0 indicates that the test and control antibodies showed the same number of exflagellations, and an inhibition index of 100 denotes that the test sample completely inhibited exflagellation. The average from multiple tests was reported to minimize the assay variation caused by the length of time that the parasites were in culture.

The human serum and red blood cells used for the gametocyte cultures, SMFA, and EXA were purchased from Interstate Blood Bank.
